# 1021. Semi-nested Molecular Method for the Diagnosis of Invasive Fungal Infections in Combat Injured

**DOI:** 10.1093/ofid/ofad500.052

**Published:** 2023-11-27

**Authors:** Graham C Ellis, Faraz Shaikh, M Leigh Carson, Erica Sercy, Laveta Stewart, Jared M Andrews, Wesley Campbell, Katrin Mende, Joseph Yabes, Ralf Bialek, David R Tribble, Brian Wickes, Anuradha Ganesan

**Affiliations:** Walter Reed National Military Medical Center, Washington, DC; Census, Rockville, Maryland; Infectious Disease Clinical Research Program, Department of Preventive Medicine and Biostatistics, Uniformed Services University of the Health Sciences, Bethesda, MD, USA, Bethesda, MD; Infectious Disease Clinical Research Program, Department of Preventive Medicine and Biostatistics, Uniformed Services University of the Health Sciences; Henry M. Jackson Foundation for the Advancement of Military Medicine, Inc., Washington, District of Columbia; Infectious Disease Clinical Research Program, Henry Jackson Foundation, Bethesda, Maryland; Landstuhl Regional Medical Center, APO, Armed Forces - AE; Walter Reed National Military Medical Center, Washington, DC; Brooke Army Medical Center, San Antonio, Texas; Brooke Army Medical Center, San Antonio, Texas; LADR Labor Dr. Kramer & Kollegen, Geesthacht, Schleswig-Holstein, Germany; Uniformed Services University of the Health Sciences, Bethesda, Maryland; UT Health San Antonio, San Antonio, Texas; Infectious Disease Clinical Research Program, USUHS; Henry M. Jackson Foundation for the Advancement of Military Medicine Inc, Bethesda, Maryland

## Abstract

**Background:**

Among combat injured, invasive fungal infections (IFI) result in significant morbidity (eg surgical amputations). Primary diagnostic methods are limited as cultures are insensitive and time delayed, and histopathology often cannot provide genus level identification needed for directed treatment. We previously evaluated a panfungal PCR (PF) assay and it was 83% sensitive and 99% specific for angioinvasive IFI (AIFI). The PF assay requires substantial expertise and cannot be easily performed at all facilities. Thus, we evaluated less resource intensive semi-nested PCR (SN) targeted to clinically relevant fungi (order *Mucorales* and genus *Aspergillus* and *Fusarium*).

**Methods:**

Formalin-fixed paraffin-embedded tissue (FFPE) specimens from a multicenter trauma IFI cohort (2009-14) were used. Cases were US military personnel injured in Afghanistan with histopathologic IFI evidence. Controls were patients with similar injury patterns and no laboratory IFI evidence (negative culture and histopathology). SN assays specific to *Mucorales* (V4/V5 regions of 18S rDNA), *Aspergillus* (P2/Asp2 regions of mitochondrial tRNA), and *Fusarium* (internal transcribed spacer [ITS]/28A regions of DNA) were compared to a PF assay amplifying the ITS2 region of rDNA and to histopathology. Performance characteristics and Cohen’s Kappa coefficients were calculated.

**Results:**

Specimens from 92 injury sites (62 subjects) were compared to control specimens from 117 injuries (101 subjects; Fig 1). We observed substantial agreement between SN and PF assays overall and for order *Mucorales* (Table 1). Moderate agreement was observed at the genus level for *Aspergillus* and *Fusarium*. When compared with histopathology, sensitivity and specificity of SN assays were 64.7% and 96.6% respectively (91.7% and 96.6% when restricted to AIFI; Table 2). No statistically significant difference in performance between the SN and PF assays was observed.

**Figure:**
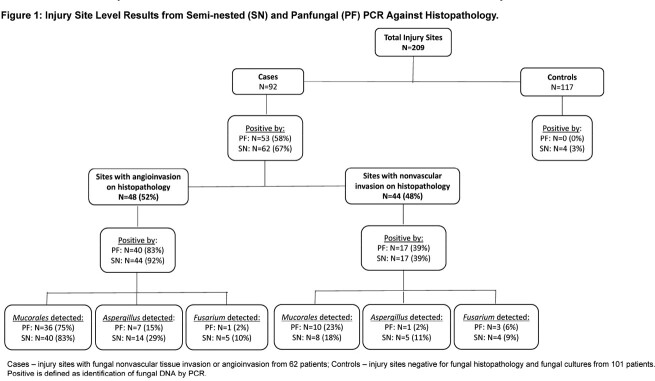

**Figure:**
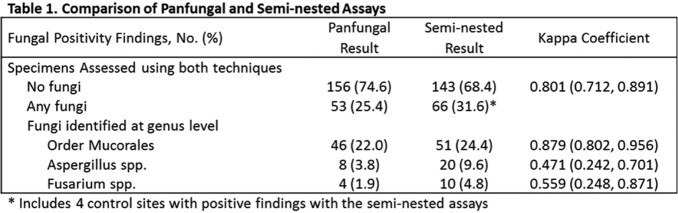

**Figure:**
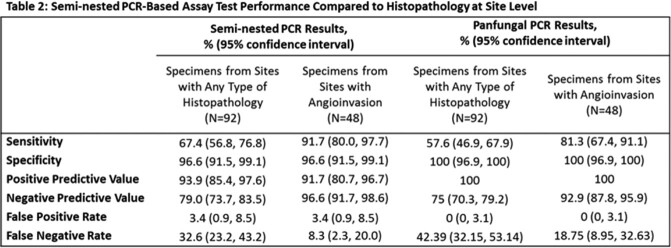

**Conclusion:**

Prior studies of SN molecular diagnostics have focused on culture-negative samples from immunocompromised patients. Our findings underscore utility of the SN approach in diagnosing IFI using FFPE samples, especially AIFI. Given the known DNA degradation with formalin fixation, sensitivity of SN assays may be higher in unfixed specimens and should be examined.

**Disclosures:**

**All Authors**: No reported disclosures

